# Adherence to statin or aspirin or both in patients with established cardiovascular disease: exploring healthy behaviour *vs*. drug effects and 10-year follow-up of outcome

**DOI:** 10.1111/j.1365-2125.2008.03212.x

**Published:** 2008-03-19

**Authors:** Li Wei, Tom Fahey, Thomas M MacDonald

**Affiliations:** Medicines Monitoring Unit (MEMO), Division of Medicine and Therapeutics, Ninewells Hospital and Medical School Dundee, UK; 1 Department of General Practice, Royal College of Surgeons in Ireland, Mercer's Medical Centre Dublin, Ireland

**Keywords:** aspirin, cardiovascular recurrence, long-term adherence, statin

## Abstract

**Aims:**

To characterize adherence in patients with established cardiovascular disease taking statins and aspirin and to estimate the effects of adherence due to health behaviour, a lack of beneficial drug effect, or both on recurrence of cardiovascular disease or all-cause mortality over 10 years.

**Methods:**

A population-based cohort study using a record-linkage database in Tayside, Scotland. Subjects with cardiovascular disease (*n* = 7657; 4185 aspirin-alone cohort, 671 statin-alone cohort and 2801 combination use cohort) were studied between 1993 and 2003. The effects of adherence on recurrence of cardiovascular disease or mortality were assessed using Poisson regression model.

**Results:**

In subjects taking both aspirin and statins, those adherent to statins but not aspirin had a lower risk of recurrence [adjusted risk ratio (RR) 0.64; 95% confidence interval 0.49, 0.82], but those adherent to aspirin but not statins has no such effect (adjusted RR 0.91; 0.72, 1.15), suggesting that adherence behaviour alone was not responsible for the beneficial effect. Within the group adherent to aspirin, ≥80% adherence to statins was associated with reduced recurrence compared with those poorly adherent (adjusted RR 0.76; 0.62, 0.94), but no such effect of aspirin was seen in those adherent to statins. Similar results were found for all-cause mortality.

**Conclusions:**

Poor health behaviour is not a sufficient explanation of adverse outcome in poorly adherent patients. Adverse outcome is more likely to be driven by foregone drug benefits.

**WHAT IS ALREADY KNOWN ABOUT THIS SUBJECT**

- Aspirin and statins are widely-used drugs in patients with cardiovascular disease.
- There is less information on healthy behaviour *vs.* drug effects.

**WHAT THIS STUDY ADDS**

- Long-term adherence to aspirin and statin treatments in patients with established cardiovascular disease has been investigated.
- Poor health behaviour is not a sufficient explanation of adverse outcome in poorly adherent patients.

## Introduction

Aspirin and statins are widely used drugs in patients with cardiovascular disease. Large clinical trials have demonstrated the benefits of these drugs on cardiovascular recurrence and mortality. However, the therapeutic effect of a drug depends not only on patients being prescribed treatment, but also on their being adherent to or compliant with the treatment. Previous adherence studies have focused on single drugs and short-term follow up (mostly ≤1 year) [1–5]. These studies have also been confounded by the notion that adherent subjects may exhibit ‘healthy behaviour’, as was seen in the placebo group of the Coronary Drug Project [6]. Thus it is not known if the outcome of poor adherence is secondary to poor health behaviour or due to the foregone efficacy of prescribed drugs.

This study aimed to investigate the long-term adherence to aspirin and statin treatments in patients with established cardiovascular disease and to estimate the effects of adherence on recurrence of cardiovascular disease or all-cause mortality. In particular, we have focused on those subjects who exhibited adherence to one drug (who presumably had good health behaviour) but not to the other.

## Methods

This study was carried out in the population of Tayside in Scotland, using the Medicine Monitoring Unit's (MEMO) record-linkage database [7]. The database contains several datasets, including all dispensed community prescriptions, hospital discharge data and other data that are linked by a unique patient identifier, the community health index number. The data have been validated and made anonymous for the purposes of research as approved by the government-appointed guardians of patient confidentiality. The project was also approved by the Tayside committee on research medical ethics.

### Study population

The study population was the population of Tayside who were resident and registered with a general practitioner between January 1993 and December 2003.

### Study subjects

The subjects were those who met the entry criteria:

1. Patients who had experienced their first cardiovascular disease hospitalization (angina, myocardial infarction, heart failure, stroke/transient ischaemic attack and peripheral vascular disease) between January 1993 and December 2001. They were identified from the Tayside hospital discharge data. Patients who had a hospitalization for cardiovascular disease before 1 January 1993 were excluded. The accuracy of diagnosis for these hospital discharge diagnosis data was about 88% [8].
2. Patients who had had at least one prescription for a statin or aspirin in the period January 1993 to December 2001. Three cohorts (i.e. the statin-alone cohort, aspirin-alone cohort and the combined statin and aspirin cohort including drug switching users) were identified from the dispensed prescribing database.
3. All subjects had at least 180 days of follow-up time in the study and at least 28 days’ follow-up time after the first prescription of aspirin or a statin.

### Study period

The study period was from January 1993 to December 2003. Patients entered the study at the date of discharge from hospital. They were followed up for use of statin or aspirin until December 2001. Cardiovascular disease recurrence or mortality outcome were followed up until December 2003.

### Statin/aspirin adherence

For each prescription for a statin we knew the strength of the tablet, the number of tablets dispensed and the instructions on how these should be taken. Thus the daily dose and the number of days’ treatment were calculated. Adherence to statin treatment was calculated as the number of days with statin supply divided by the total number of days from the first prescription for a statin to the end of study or the end of 2001. If a patient collected more drugs than they had been directed to use, the percentage of adherence was >100%, but we classified these subjects as having maximum adherence. Good adherence was defined as ≥80% adherence and partial adherence was defined as <80% adherence. The 80% cut-off point was used because it is used conventionally in clinical trials of safety and efficacy that have been used to support a new drug registration [9]. Adherence to aspirin was calculated in the same way as statin adherence.

### Study outcomes

The outcomes of the study were adherence to statin or aspirin treatment during the follow-up period and cardiovascular disease recurrence (defined as hospitalization for recurrent cardiovascular disease or cardiovascular death) or all-cause mortality until December 2003.

### Statistical analysis

Data were summarized as mean (SD) for continuous variables and number of subjects (%) for categorical variables. χ^2^ and *t*-tests were performed to determine significant differences. Cochran–Armitage trend tests were also performed if there were more than two categorical variables. The Poisson regression model was used to analyse the time to hospitalization for recurrent cardiovascular disease or all-cause mortality, giving results in terms of risk ratios (RR). Analyses were carried out univariately and multivariately. Multivariable analyses adjusted for age, sex, socio-economic deprivation, calendar year of entry to the study, presence of diabetes mellitus at baseline (defined as on antidiabetic treatment), other cardiovascular drug prescriptions during the follow-up including angiotensin converting enzyme inhibitors, β-blockers, calcium channel blockers, anticoagulant, cardiac glycosides, diuretics and nitrates, and amount of co-prescribing of cardiovascular drugs. To minimize confounding by the ‘healthy adherer’ effect (i.e. an adherence effect on outcome attributable to the adoption of healthier lifestyles that accompany adherence behaviours) [2], a prespecified subgroup analysis was performed within patients who were on both statins and aspirin treatments, contrasting those who were adherent to statins but not aspirin with those adherent to aspirin but not statins. Interaction between aspirin adherence and nonsteroidal anti-inflammatory drug use during follow-up was adjusted for in those adherent to statins, as a previous study has shown that ibuprofen may interact with the cardioprotective effects of aspirin [10]. All statistical analyses were carried out using SAS version 8.0 (SAS Institute, Cary, NC, USA).

## Results

**Figure 1:**
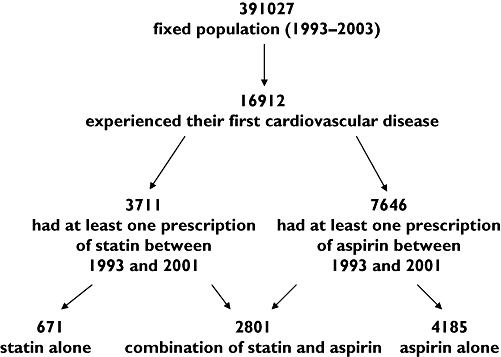
Flowchart of the patients in the three cohorts

There were 671 patients in the statin-alone cohort, 4185 in the aspirin-alone cohort and 2801 in the combination use cohort. Figure 1 shows the flow chart of subjects entered in each cohort.

### Drug adherence and characteristics of patients

Table 1 shows the distribution of adherence in the different groups. The percentages of good adherence to drug treatment were 60.0% for the aspirin-alone group, 64.5% for the statin-alone group and 76% for the combination use group (*P* < 0.01) during an average follow-up time of 4.7 years.

**Table 1:** Adherence to drug treatment in the different cohorts

	Adherence to drug treatment	
	<80% *n* (%)	≥80% *n* (%)
**Aspirin-alone cohort**	1673 (40.0)	2512 (60.0)
**Statin-alone cohort**	238 (35.5)	433 (64.5)
Combination use cohort*	464 (24.0)	1471 (76.0)

* Only included patients who had <80% adherence to statins and <80% adherence to aspirin or ≥80% adherence to aspirin and ≥80% adherence to statins.

### Descriptive statistics on adherence levels

The proportion of patients who had good adherence (≥80% adherence) in the statin-alone or combination cohorts decreased from the end of year 1 to the end of year 8 (Table 2), but was similar in the aspirin-alone cohort. Table 3 shows the characteristics of patients in the different adherence groups. There were significant differences in age and social deprivation between the good adherence and partial adherence patients. Subjects in the good adherence group were older and less deprived compared with the partial adherence group.

**Table 2:** Adherence to drug treatment by follow-up time in patients with cardiovascular disease

	Aspirin-alone group		Statin-alone group		Combination use group			
	*n* = 4185		*n* = 671		Aspirin (*n* = 2801)		Statin (*n* = 2801)	
Follow-up time for drug use, years	No. of patients remained at the end of each year	% of patients with good adherence	No. of patients remained at the end of each year	% of patients with good adherence	No. of patients remained at the end of each year	% of patients with good adherence	No. of patients remained at the end of each year	% of patients with good adherence
**1**	3269	57.8	454	59.5	2281	65.6	2167	66.5
**2**	2361	56.1	296	55.7	1716	64.5	1503	65.1
**3**	1751	54.0	178	51.2	1234	62.6	971	62.7
**4**	1292	54.6	100	51.0	867	61.0	585	63.1
**5**	887	55.7	47	51.1	592	58.6	327	62.1
**6**	564	55.1	27	37.0	375	58.7	132	62.1
**7**	311	53.1	10	30.0	220	56.8	31	61.3
**8**	140	57.9	6	33.0	91	61.5	5	60.0

**Table 3:** Distribution of characteristics by adherence in the different cohorts

	Aspirin alone		Statin alone		Combination			
	*n* = 4185		*n* = 671		Aspirin (*n* = 2801)		Statin (*n* = 2801)	
	<80% *n* = 1673	80∼100% *n* = 2512	<80% *n* = 238	80∼100% *n* = 433	<80% *n* = 911	80∼100% *n* = 1890	<80% *n* = 883	80∼100% *n* = 1918
**Sex Male**	858 (51.3)	1253 (49.9)	154 (64.7)	251 (58.0)	540 (59.3)	1093 (57.8)	504 (57.1)	1129 (58.9)
** Female**	815 (48.7)	1259 (50.1)	84 (35.3)	182 (42.0)	371 (40.7)	797 (42.2)	379 (42.9)	789 (41.1)
Age group**								
** <40**	20 (1.2)	14 (0.6)	13 (5.5)	5 (1.2)	22 (2.5)	27 (1.4)	21 (2.3)	28 (1.5)
** 40–49**	97 (5.8)	56 (2.2)	50 (21.0)	71 (16.4)	109 (12.3)	164 (8.6)	129 (14.2)	144 (7.6)
** 50–59**	192 (11.5)	231 (9.2)	86 (36.1)	132 (30.5)	228 (25.8)	475 (24.8)	259 (28.4)	444 (23.5)
** 60–69**	375 (22.4)	535 (21.3)	45 (18.9)	128 (29.5)	283 (33.2)	742 (38.7)	292 (32.1)	743 (39.3)
** 70–79**	523 (31.3)	924 (36.8)	33 (13.9)	80 (18.5)	194 (22.0)	452 (23.6)	182 (20.0)	464 (24.6)
** ≥80**	466 (27.9)	752 (29.9)	11 (4.6)	17 (3.9)	37 (4.2)	58 (3.0)	28 (3.1)	67 (3.5)
Deprivation category†*								
** 1 least deprived**	88 (5.3)	180 (7.2)	19 (8.0)	43 (9.9)	55 (6.2)	132 (6.9)	54 (5.9)	133 (7.0)
** 2**	262 (15.7)	430 (17.2)	44 (18.5)	78 (18.0)	134 (15.2)	333 (17.4)	143 (15.7)	324 (17.2)
** 3**	440 (26.3)	679 (27.1)	52 (21.9)	107 (24.7)	209 (23.7)	485 (25.3)	223 (24.5)	471 (24.9)
** 4**	324 (19.4)	473 (18.9)	36 (15.1)	92 (21.3)	158 (17.9)	383 (20.0)	166 (18.2)	375 (19.9)
** 5**	214 (12.8)	297 (11.9)	32 (13.5)	40 (9.2)	101 (11.5)	200 (10.4)	111 (12.2)	190 (10.1)
** 6,7 most deprived**	343 (20.5)	445 (17.8)	55 (23.1)	73 (16.9)	225 (25.5)	384 (20.0)	213 (23.4)	396 (21.0)

Data are numbers and % unless stated;

† excluding missing data.

* *P* < 0.05;

** *P* < 0.01.

### Relationship between adherence and cardiovascular disease recurrence

The effects of adherence to aspirin alone, statin alone and the combination were assessed for outcome of recurrence of cardiovascular disease. The number of cardiovascular events in each group was 2075, 166 and 726, respectively. The rates of events were 53 [95% confidence interval (CI) 45, 60], 112 (95% CI 107, 116) and 14 (95% CI 13, 15) per 1000 person-years, respectively. Table 4 shows the results of both univariate and multivariate Poisson regression analysis for recurrence of cardiovascular disease.

**Table 4:** Univariate and multivariate relative risks for recurrence of cardiovascular disease in the different cohorts

Outcome predictor	Univariate		Multivariate§	
	RR	95% CI	RR	95% CI
***Aspirin-alone cohort***				
**Adherence to aspirin (%)**				
** <80**	1.00		1.00	
** 80–100**	1.14	1.04, 1.24	1.08	0.97, 1.21†
***Statin-alone cohort***				
**Adherence to statin (%)**				
** <80**	1.00		1.00	
** 80–100**	0.83	0.61, 1.12	0.66	0.47, 0.91*
***Combination cohort***				
**Adherence to both (%)**				
** Both <80%**	1.00		1.00	
** Statin ≥80% and aspirin <80%**	0.67*	0.52, 0.86	0.64	0.49, 0.82**
** Statin <80% and aspirin ≥80%**	0.93	0.74, 1.18	0.91	0.72, 1.15
** Both ≥80%**	0.82*	0.68, 0.99	0.69	0.56, 0.84**

§ Adjusted for age, gender, social deprivation, calendar year, diabetes mellitus, cardiovascular drug use during follow-up and number of cardiovascular prescriptions.

† Also adjusted for interaction between aspirin adherence and nonsteroidal anti-inflammatory drug use.

* *P* < 0.05;

** *P* < 0.01.

There was no significant difference in cardiovascular disease recurrence between good and partial adherence in the aspirin-alone group (adjusted RR 1.08, 95% CI 0.97, 1.21). There was a lower risk of recurrence of cardiovascular disease in the statin-alone group (adjusted RR 0.66, 95% CI 0.47, 0.91). Compared with those who were not adherent to both statins and aspirin (i.e. statin adherence <80% and aspirin adherence <80%), patients with ≥80% adherence to both statins and aspirin or patients with ≥80% adherence to statins and <80% adherence to aspirin had a lower risk of recurrence of cardiovascular disease (adjusted RR 0.69, 95% CI 0.56, 0.84; and 0.64, 95% CI 0.49, 0.82, respectively). However, those adherent to aspirin but not to statins had no such lower risk of cardiovascular disease recurrence.

To explore the differential effect on cardiovascular disease recurrence in the combination cohort we examined the differential effects of aspirin and statin adherence (Table 5). In those subjects adherent to aspirin, good adherence to statins predicted a better outcome (adjusted RR 0.76, 95% CI 0.62, 0.94). However, in subjects adherent to statins, good adherence to aspirin did not predict a better outcome (adjusted RR 1.03, 95% CI 0.76, 1.39).

**Table 5:** Univariate and multivariate relative risks for recurrence of cardiovascular disease and all-cause mortality in the good adherence subgroup

	Univariate		Multivariate§	
Outcome predictor	RR	95% CI	RR	95% CI
**Cardiovascular recurrence**				
***Within statin adherence*≥*80% subgroup***				
**Adherence to aspirin (%)**				
** <80**	1.00		1.00	
** 80–100**	1.22	0.98, 1.52	1.03	0.76, 1.39†
***Within aspirin adherence*≥*80% subgroup***				
**Adherence to statin (%)**				
** <80**	1.00		1.00	
** 80–100**	0.88	0.72, 1.08	0.76	0.62, 0.94*
**All-cause mortality**				
***Within statin adherence*≥*80% subgroup***				
**Adherence to aspirin (%)**				
** <80**	1.00		1.00	
** 80–100**	1.22	0.80, 1.84	0.73	0.44, 1.22†
***Within aspirin adherence*≥*80% subgroup***				
**Adherence to statin (%)**				
** <80**	1.00		1.00	
** 80–100**	0.80	0.56, 1.15	0.72	0.50, 1.05

§ Adjusted for age, gender, social deprivation, calendar year, diabetes mellitus, cardiovascular drug use during follow-up and number of cardiovascular prescriptions.

† Also adjusted for interaction between aspirin adherence and nonsteroidal anti-inflammatory drug use.

* *P* < 0.05; ** *P* < 0.01.

### Relationship between adherence and all-cause mortality

The numbers of deaths during the follow-up were 1293 in the adherence to aspirin alone group, 61 in the statin-alone group and 222 in the combination group. The mortality rates were 17 (95% CI 13, 21) per 1000 person-years for the statin-alone group, 51 (95% CI 48, 54) for the aspirin group and two (95% CI 2, 3) for the combination use group. The adjusted RRs for the good adherence group compared with the partial adherence group were 0.85 (95% CI 0.75, 0.97) for the aspirin-alone group and 0.72 (95% CI 0.42, 1.24) for the statin-alone group. Compared with the group of <80% adherence for both statins and aspirin, the adjusted RRs were 0.68 (95% CI 0.57, 0.87) for the group of ≥80% adherence to statins and <80% adherence to aspirin, 0.93 (95% CI 0.73, 1.17) for the group of <80% adherence to statins and ≥80% adherence to aspirin and 0.72 (95% CI 0.59, 0.88) for the group of ≥80% adherence to both statins and aspirin.

## Discussion

Our study is the first to examine the effects of prolonged dual adherence to aspirin and statins in patients with cardiovascular disease in the setting of primary care. Overall, patients who were taking both treatments had better adherence than those who were prescribed single drug treatments. Good adherence was associated with better outcome in patients who were taking both treatments when compared with partial adherence.

### Adherence to drug treatment

The benefits of cardiovascular disease prevention depend upon high-risk patients achieving good adherence to medical regimens of proven efficacy [9]. The better adherence found in patients who took both aspirin and statin treatment may reflect disease severity, i.e. patients who had more severe conditions had better adherence to medication than those who had less severe conditions. Adherence to statin treatment prescribed outside of clinical trials is poor [11]. Our study has shown that long-term adherence in cardiovascular patients is suboptimal, especially in patients who were on single drug treatment. However, the rates of adherence to statin and aspirin treatment in our study were higher than in other studies [4, 12–15]. Deprivation was also associated with adherence in our study, and this was supported by another study in the USA that showed that lower socio-economic status was linked to lower adherence [16].

### Effect of adherence on cardiovascular recurrence or mortality

Previous studies have shown that compliance with cardiovascular drug treatment improves outcome [17, 18]. Simpson and colleagues recently conducted a meta-analysis of the association between adherence to drug therapy and mortality [19]. However, most studies have focused on single drug effects. There are no reports of long-term adherence to combinations of drug treatment. In the present study, the beneficial effect of good adherence was found in patients who were on both drug treatments and in patients who were taking statin treatment. Although there was a lower point estimate (0.64 *vs.* 0.69) for outcome in patients who were adherent to statin but not aspirin compared with those who were adherent to both, the 95% CIs overlapped, and thus this difference could be due to chance alone.

An important finding in our study is that we provide data on the ‘disconnect’ between adherence behaviour and outcome. Thus, those patients adherent to aspirin (who presumably had good healthy behaviour) but who were not adherent to statins had a worse outcome than those who were adherent to statins. This provides good evidence that behaviour alone cannot account for these effects of adherence. Interestingly, in those adherent to statins, adherence to aspirin was not associated with better outcome. If the better outcome with good adherence was largely due to behavioural issues then aspirin adherence would be strongly linked to good outcome. In practice, this is not the case. There may also be unmeasured confounders that we could not control for in relation to aspirin adherence and the resulting beneficial effect of aspirin therapy. Alternatively, reduced adherence may not be as important with aspirin therapy. For example, the Physicians Health Study detected a beneficial effect of aspirin given every second day (i.e. 50% adherence) [20].

### Strengths and limitations

The strengths of the present study are: (i) it was a population-based cohort design with complete follow-up over the study period. This approach allowed a ‘real-world’ population to be studied representing all socio-economic groups and within a universal healthcare coverage scheme [21]. Unlike clinical trials, which focus on highly selected patients [81–100% men and the young (mean age 55–59 years)][22], population-based record-linkage studies allow real-world populations to be studied. In our study, about 46% of patients were women and the average age of patients was 67.5 years; (ii) MEMO collects only dispensed prescribing and so primary noncompliance is eliminated [23]; (iii) our study shows the details of long-term adherence and its effect on outcome, especially those of concomitant drug treatment, whereas other studies have focused on single drug effects and did not account for multiple drug effect.

Our study has some limitations. First, MEMO does not have information on certain risk factors such as lifestyle, i.e. smoking, alcohol and exercises. These limitations are not unique to MEMO's record-linkage database; other databases also do not have routinely collected information on lifestyle or drug indication [24]. However, we were able to adjust for social deprivation, which is a marker of both poor adherence and poor health behaviour [25]. Second, we may have underestimated the intention-to-treat with statins and aspirin in the present study, as adherence to treatment was based on the dispensing of prescriptions after discharge from hospital. Thus, we could not distinguish between people who were not adherent to prescriptions that were written but were not redeemed at pharmacies, those who had prescriptions that were written but not collected from the practice and those who were never prescribed statins. Third, we were not able to take account of the effect of aspirin that was purchased over-the-counter (OTC). However, our previous study [26] has shown that there were hardly any aspirin prescriptions purchased OTC, and therefore any bias derived from the OTC would be minimal and would not be able to change our main results. We assumed that if a prescription was filled then patients would adhere to treatment, but we had no way of knowing whether or not subjects actually took the pills. However, this problem is not unique, and in fact this limitation applies to the vast majority of studies, including randomized controlled trials.

In conclusion, patients who were taking both statin and aspirin treatments had better adherence than those who were on single drug treatment. Good adherence to both statins and aspirin treatment was associated with lower risks of recurrence of cardiovascular disease and all-cause mortality. Although adherence to statins predicted a good outcome, adherence to aspirin as a group did not. In subjects adherent to one group but not the other, no consistent effect compatible with a ‘healthy behaviour’ effect was seen, so we conclude that a major component of the adverse outcome seen with poor adherence are the foregone benefits of drug treatment.

This study was funded by the UK Medical Research Council. L.W. holds a Special Training Fellowship in Health Services and Health of the Public Research award from the UK Medical Research Council.

*Competing interest*: L. Wei and T. Fahey. T. M. MacDonald has received honoraria for lectures and advisory board fees from AstraZeneca, kaiser Permanente, Novartis, Pfizer, Recordati, Takeeda and Speedel.
